# Why a successful task substitution in glaucoma care could not be transferred from a hospital setting to a primary care setting: a qualitative study

**DOI:** 10.1186/1748-5908-8-14

**Published:** 2013-01-25

**Authors:** Kim M Holtzer-Goor, Thomas Plochg, Hans G Lemij, Esther van Sprundel, Marc A Koopmanschap, Niek S Klazinga

**Affiliations:** 1 Institute for Health Policy and Management/Institute for Medical Technology Assessment, Erasmus University Rotterdam, Rotterdam, the Netherlands; 2 Academic Medical Center (AMC), University of Amsterdam, Amsterdam, the Netherlands; 3 Glaucoma service, The Rotterdam Eye Hospital, Rotterdam, the Netherlands; 4 Glaucoma Research Unit, The Netherlands and Rotterdam Ophthalmic Institute, Rotterdam, the Netherlands

**Keywords:** Diffusion of innovation, Access to health care, Quality of health care

## Abstract

**Background:**

Healthcare systems are challenged by a demand that exceeds available resources. One policy to meet this challenge is task substitution-transferring tasks to other professions and settings. Our study aimed to explore stakeholders’ perceived feasibility of transferring hospital-based monitoring of stable glaucoma patients to primary care optometrists.

**Methods:**

A case study was undertaken in the Rotterdam Eye Hospital (REH) using semi-structured interviews and document reviews. They were inductively analysed using three implementation related theoretical perspectives: sociological theories on professionalism, management theories, and applied political analysis.

**Results:**

Currently it is not feasible to use primary care optometrists as substitutes for optometrists and ophthalmic technicians working in a hospital-based glaucoma follow-up unit (GFU). Respondents’ narratives revealed that: the glaucoma specialists’ sense of urgency for task substitution outside the hospital diminished after establishing a GFU that satisfied their professionalization needs; the return on investments were unclear; and reluctant key stakeholders with strong power positions blocked implementation. The window of opportunity that existed for task substitution in person and setting in 1999 closed with the institutionalization of the GFU.

**Conclusions:**

Transferring the monitoring of stable glaucoma patients to primary care optometrists in Rotterdam did not seem feasible. The main reasons were the lack of agreement on professional boundaries and work domains, the institutionalization of the GFU in the REH, and the absence of an appropriate reimbursement system. Policy makers considering substituting tasks to other professionals should carefully think about the implementation process, especially in a two-step implementation process (substitution in person and in setting) such as this case. Involving the substituting professionals early on to ensure all stakeholders see the change as a normal step in the professionalization of the substituting professionals is essential, as is implementing the task substitution within the window of opportunity.

## Background

Healthcare systems across many countries face a challenge in responding to growing demands for physicians’ and nurses’ care with increasing limitations on human and financial resources [1,2]. Extrapolations have shown that the number of physicians cannot keep pace with the growth in demand caused by ageing populations, enhanced societal expectations, and new diagnostic technologies [3-6].

One option to cope with workforce shortages is task substitution, which can be defined as devolving clinical responsibilities to lesser or more narrowly-trained health professionals with or without supervision. [7] Task substitution can be realised with people (*e.g.*, a diabetes nurse practitioner substitutes for an internist of the same department), settings (*e.g.*, a primary care neurologist substitutes for a hospital-based neurologist), or both (*e.g.*, primary care midwives substitute for hospital-based gynaecologists). Research has shown that task substitution may improve the quality of care [8-23] and reduce costs because substitutes’ fees are lower [24]. Strong evidence for cost savings is lacking however, perhaps because physician-substitutes perform additional tasks [25] or are less productive [26], offsetting potential cost savings. Furthermore, the successful implementation of task substitution is at least partially influenced by contextual factors, such as local stakeholder interests [27-30], power positions [31,32], and the structure of the healthcare system, including its financing [33]. It therefore seems worthwhile to broaden the scope of evaluation and include the professional, organizational, financial, and political contexts within which task substitution is implemented [34].

We explored a task-substitution project involving glaucoma care at the Rotterdam Eye Hospital (REH) in the Netherlands (see Additional file 1 for background information). Our research question was two-tiered: how do stakeholders perceive the feasibility of implementing task substitution of person (from ophthalmologists to allied health professionals) and setting (from a hospital to a primary care setting), and what are their supporting and opposing arguments?

### Historical background

Our case study was not the first initiative of the REH to cooperate with primary care optometrists working in optical shops in the Rotterdam area (later united in a Collective of Optometrists in Rijnmond Region – OCR). The first initiative, started in 1997, led to the Transmural Glaucoma project (TG-project), a preliminary person and setting task substitution project in 1999. One part of the project consisted of primary care optometrists supplementing glaucoma specialists in monitoring glaucoma patients by means of GDx-technology (Carl Zeiss Meditec, Dublin, CA, USA), an imaging tool to assess (damage to) the nerve fibre layer. REH glaucoma patients were referred to a local primary care optometrist for three additional tests between two visits to the hospital-based glaucoma specialist. It appeared difficult to convince patients to visit a primary care optometrist; only twelve of the twenty patients (60%) actually did so [35,36]. Nor were glaucoma specialists eager to participate because they could have referred more patients.

Given the results, the REH management suspended the project and initiated an intermediate step of task substitution of person only. The REH set up a Glaucoma Follow-up Unit (GFU) in the hospital and evaluated its impact through an RCT [37]. The GFU was staffed by a hospital optometrist and ophthalmic technicians who monitored the glaucoma patients according to a working protocol (Additional file 1). Four years after the successful implementation of the GFU [37], REH managers began the step of substituting primary care optometrists in optical shops for the GFU, as in the original plan (substitution of person and setting).

## Methods

An in-depth single case study evaluation was carried out from September 2007 to August 2008 using semi-structured face-to-face interviews and a document review to explore the feasibility of using primary care optometrists in optical shops as substitutes for in-hospital GFU employees.

### Sampling and recruitment

#### Semi-structured interviews

We selected 27 participants based on role, profession, and organization, thereby drawing on three sampling strategies. First, we included all four REH glaucoma specialists, five GFU employees, and the responsible hospital managers (CEO, CFO, manager of the Eye Care Network, and the advisor concerned with optometry relations). Second, we used convenience sampling to identify five primary care optometrists and two representatives of the major health insurers in the Rotterdam region. We contacted the Dutch Healthcare Authority to identify potential participants. Third, we randomly selected five patients who had participated in the GFU study, taking care that the sample included patients with only a risk factor for glaucoma as well as stable glaucoma patients and employed as well as unemployed patients. One patient was selected because of his function as chairman of the Dutch Glaucoma Patient Association. The sample is shown in Table 1.

**Table 1:** Interviewed stakeholders

**Staff Rotterdam Eye Hospital**				
Respondent	Position			Interviewers
1	CEO Rotterdam Eye Hospital			ES & TP
2	CFO Rotterdam Eye Hospital			KHG & TP
3	Manager of the Eye Care Network			KHG & ES
4	Advisor concerned with optometry relations			ES
5	Glaucoma specialist, Rotterdam Eye Hospital			ES
6	Glaucoma specialist, Rotterdam Eye Hospital			ES & TP
7	Glaucoma specialist, Rotterdam Eye Hospital			KHG
8	Glaucoma specialist, Rotterdam Eye Hospital			KHG
9	Ophthalmic technician, Rotterdam Eye Hospital			KHG
10	Optometrist, Rotterdam Eye Hospital			ES
11	Ophthalmic technician, Rotterdam Eye Hospital			ES
12	Ophthalmic technician, Rotterdam Eye Hospital			KHG
13	Ophthalmic technician, Rotterdam Eye Hospital			KHG
**Primary care optometrists**				
Respondent	Self-employed / optical chain	Participant OCR		Interviewers
14	Self-employed	Yes		KHG
15	Self-employed	Yes		KHG
16	Self-employed	Yes		ES
17	Optical chain	No		TP
18	Self-employed	Yes		TP
19	Optical chain	Yes		ES
**Patients**				
Respondent	Travelling distance to REH (in kilometres)	Working status	Severity of the disease	Interviewers
20	21	Employed	Risk factor	TP
21	19	Unemployed	Glaucoma	ES
22*	75	Employed	Glaucoma	TP
23	14	Employed	Risk factor	KHG
24	18	Unemployed	Suspect	KHG
**Health insurers / The Dutch Healthcare Authority**				
Respondent	Position			Interviewers
25	Health insurer (Health insurance only)			ES & MK
26	Health insurer (All kinds of insurances)			KHG & TP
27	Senior policy advisor of The Dutch Healthcare Authority			KHG

* chairman of the Dutch Glaucoma Patient Association; REH = Rotterdam Eye Hospital.

#### Document review

Relevant policy and administrative documents were continually collected during the study period (2004 to 2009). Their sources were suggested by participants or found on the internet and websites of relevant stakeholders. EvS and KHG selected the documents when they considered any part of them relevant to the research question. Selected documents included public information, official policy reports, minutes of meetings, and working documents.

#### Procedure

Two researchers conducted the first six interviews together because it allowed them to give each other feedback on the interviewing process. The remaining interviews were done by one of four researchers (KHG, EvS, TP, MK). We developed a topic list (Additional file 2) based on the research question to guide the interviews, which contained open questions that left room for participants to expand and clarify their answers. Moreover, they had the opportunity to express their opinions and to share what was important to them concerning the feasibility of transferring glaucoma care to primary care optometrists. The interviews took approximately one hour each, were audio recorded, and later transcribed verbatim.

#### Analytic approach

The transcripts of interviews and documents were inductively analysed for the respondents’ views regarding the feasibility of the task substitution. We thereby used an analytic approach, drawing on three theoretical perspectives.

First, we used sociological theories on professionalism to explore professionals’ views and interprofessional dynamics. Professions are sociologically defined as groups of institutions that permit the members of an occupation to make a living while controlling their own work [38]. From such a sociological perspective, implementing task substitution is not a technical solution, but rather a social process affecting the professional status of those involved [38-42]. Key to our analysis was how hospital-based glaucoma specialists, primary care optometrists, and GFU employees viewed the feasibility of the desired task substitution, and how it related to opportunities for or threats to controlling their work.

Second, we applied management theories to explore managerial rationales and views underpinning the desired substitution of tasks. Research shows that evidence-based interventions to improve quality of care are not automatically implemented and returns on investments or so-called ‘business cases for quality’ are often absent or too small to be effective [43]. Moreover, an organizational infrastructure should be in place to support the innovation. Here, we explored how the respondents viewed the business case for the task substitution and whether they thought an appropriate infrastructure was in place.

Third, applied political analysis was used to map the interests and power positions of each stakeholder involved [31]. Their interests regarding the task substitution (supporting or opposing) together with their power positions and willingness to use them structure the political feasibility of successful implementation of task substitution.

#### Ensuring rigour

We used different strategies to monitor and enhance the rigour of data collection, analysis, and validity. First, we validated key findings by data triangulation. Data collected from different sources (semi-structured interviews, document analysis, and literature) and researchers were compared to verify specific findings. Second, we sought feedback from senior and other researchers (peer review; HL, MK, NK), who critically appraised the research process and earlier drafts of the article. Third, reflexivity of the main researchers (KHG, TP, and EvS) was applied to rule out threats to validity due to reactivity and researcher bias.

## Results

Our threefold data analysis showed that it is currently not feasible to implement task substitution in this particular case. Respondents’ narratives revealed that the intermediate establishment of a suitable hospital setting (the GFU) in 2004 pre-empted the original sense of implementation urgency. Nor did the professionals (ophthalmologists and GFU employees) consider the shift to shop setting a positive step towards further professionalization. An unclear return on investment did not help matters. Last, the power positions of reluctant key stakeholders were strong enough to block the implementation of task substitution from the hospital to the primary care setting. Table 2 contrasts the initial assumptions of the stakeholders with the perceived feasibility as expressed by the participants.

**Table 2:** Stakeholder positions concerning the task substitution of person and setting before and after GFU establishment

		**Theory 1: closed window**	**Theory 2: unclear returns on investments**	**Theory 3: power position - level interest**
**Before GFU establishment**	Glaucoma Specialists	High workload, increasing demand for glaucoma care.	Workload release, decreasing the waiting list, more challenging work.	High power position and high interest for a successful task substitution.
	Management REH	Increased competition on volume.	Increase in volume of (new) patients.	Medium power position and high interest for a successful task substitution.
	Primary care optometrists	Competition with optical chains, chance to professionalize.	Increase in volume of (new) patients.	Low power position and high interest for a successful task substitution.
	Patients	Were not involved at the start.	More flexible appointments and more time per appointment.	Medium power position and medium interest for a successful task substitution.
	Dutch Health Care Authority / Health insurers	Were not involved at the start.	Care would possibly become less expensive	High power position and unclear interest for a successful task substitution.
	GFU employees	Were not involved at the start.		Low power position and low interest for a successful task substitution.
**After GFU establishment**	Glaucoma Specialists	Release of workload due to GFU.	The establishment of the GFU already fulfilled their goals.	Reduction of interest for a successful task substitution.
	Management REH	Better alternative was found through cooperation with optical chain.	Disappointing increase in volume due to cooperation with OCR. Alternative was found.	Reduction of interest for a successful task substitution.
	Primary care optometrists	Cooperation remained on the same level.	Increase in new patients differed among optometrists.	Reduction of chance to strengthen relationship with glaucoma specialists
	Patients	GFU resulted in more time per patient, and care in a familiar setting.		Reduction of interest for a successful task substitution.
	NZA / Health insurers	Quality of care in GFU was good.		No changes in interest due to establishment of GFU.
	GFU employees	Improved relationship with glaucoma specialists and more satisfying work.	The consequences of starting task substitution for the GFU were unclear.	Increase of power position.

### Closed window of opportunity

The analysis from the professionalization perspective revealed that the window of opportunity for task substitution closed with an intermediate step, *i.e.*, establishing the GFU. In the late 1990s, waiting lists (demand pressures), new GDx technology, and competition from ophthalmologists working in private clinics all favoured the task substitution of both person (from ophthalmologists to optometrists) and setting (from hospital to primary care). Professional dynamics, however, impeded the twofold implementation strategy.

The first step (substitution within the hospital setting – the GFU) eased the pressures on the glaucoma specialists, and the GFU employees enjoyed their work. As a consequence, the glaucoma specialists and GFU employees no longer supported the final step, which was implicitly reflected in the debate on the expertise of primary care optometrists.

Optometrists’ subtle and constructive views confirmed that quality of care was perceived to be the most important factor for the feasibility of the task substitution. All six primary care optometrist-interviewees were convinced of their capability to monitor stable glaucoma patients (Table 3) because during the TG-project and the TOZ-project (transmural eye care for all indications) some participating OCR optometrists gained experience in screening patients and strengthened their relationships with REH ophthalmologists, and they were trained to detect pathological abnormalities of the eye.

**Table 3:** Quotations ‘closed window of task substitution’

**Stakeholder**	**Quotation**
Primary care optometrists:	
·	As an optometrist you have done everything during your training, you have seen all the abnormalities, you have read and learned about them, and you graduated. (Respondent 14)
·	Considering our experience in the TOZ project (transmural eye care), in my opinion, we are capable of providing, without any problems, part of the care for stable glaucoma patients and patients with a risk factor for glaucoma. (Respondent 15)
·	We don’t see enough glaucoma patients to monitor them. Even though it can occasionally occur, I do think that we need to get more practical experience of these patients on a daily basis. If we start monitoring patients, we have to know how the eye hospital wants it to be done, how they do it, and what they exactly want to know. This can only be achieved through training. By watching glaucoma specialists at work. (Respondent 19)
Glaucoma specialists and GFU employees:	
·	To me, the GFU is a good system because I do have some idea of the quality being delivered. And I think that is essential to know. I am not in favour of transferring this care to optometrists who work outside of the REH, because then I'm not sure what the quality of their care will be. (Glaucoma specialist, respondent 7)
·	Unfortunately, we have had quite some bad experiences with a number of primary care optometrists. A small number, but quite bad experiences. They were playing at being doctors, without having the knowledge. That’s what I’m concerned about. (Glaucoma specialist, respondent 8)
·	I still see the quality of care of these optometrists on a weekly basis (TG project), and I think that this group is not suitable for monitoring these patients. I still see too many assessments, where they say, there's nothing wrong, and where I think: well there is definitely something wrong. (GFU employee, respondent 9)

Some primary care optometrists indicated, however, that they would like to have more routine monitoring of glaucoma patients to bolster their initial education (Table 3). In response, the REH organised training guided by glaucoma specialists for the optometrists participating in the TG-project. Optometrists interned for several days, studied a textbook, and were tested before they could participate. Despite this training, glaucoma specialists and GFU employees were, due to their experience during the TG-project in the late 1990s, not convinced of the primary care optometrists’ expertise. The glaucoma specialists doubted whether the quality of care delivered by optometrists would be comparable to GFU employees despite the additional education. The glaucoma specialists were furthermore eager to check the GFU employees’ quality of care in the hospital setting and feared losing control over their patients in an outpatient setting (Table 3).

Having the GFU staffed by hospital optometrists instead of primary care optometrists further reduced the likelihood that the task substitution to primary care optometrists would succeed. Besides eliminating the sense of urgency for task substitution of person and setting, establishment of the GFU strengthened the bond between the glaucoma specialists and GFU employees, closing the window of opportunity for task substitution of person and setting.

### Unclear returns on investments

The stakeholders doubted whether the monitoring of stable glaucoma patients by primary care optometrists would still be financially interesting, *i.e.*, the return on investment was unclear (see Table 4). The high workload of the glaucoma specialists, which sparked the initiative, was significantly reduced by the establishment of the GFU in 2004. Increased capacity made it possible to lift the ban on accepting new glaucoma patients, which resulted in a 23% increase from 2004 to 2008. Two glaucoma specialists indicated that the increase in capacity eliminated the pressure to further pursue task substitution. Moreover, one underlying key assumption proved to be untrue. The REH management assumed that the task substitution would increase capacity and inflow of new patients. But in 2008 the primary care optometrists within REH’s eye care network were responsible for only 1% of the new patient inflow. The collective OCR organization was furthermore rudimentary: the optometrists were mostly self-employed, and could not be easily approached as a group. For both reasons, the REH started collaborating with a large optical chain to ensure a steady inflow of new patients.

**Table 4:** Quotations ‘Unclear returns on investments’

**Stakeholder**	**Quotation**
Glaucoma Specialists:	
·	I: But it is getting busier with glaucoma patients, and you cannot discharge everyone. R: That's why we created this system, the GFU. I: Do you think that is enough? R: I think so. (Respondent 6)
·	If the pressure, the number of patients at the clinic increases, and we have to announce waiting lists again or limit the number of patients at some point, then it will not be beneficial to the quality of care. Then we'll have to do something like that [task substitution], we'll have to go down that road. (Respondent 8)
REH Management:	
·	What we do is, we move the chronic patients. Those patients are not financially attractive, not for the partnership either. So to make it financially attractive, we need to see new patients, we must get referrals. (Respondent 1)
·	Primary care optometrists only send 1% of our referrals. So we need to arrange the other referral channels. (Respondent 3)
Primary Care Optometrists:	
·	But when an optical chain joins, it makes us less unique. And as an independent optical shop, we take optometry very seriously. (Respondent 16)
·	Yes, there are customers who come to our shop, even if I do not know them personally… I have not seen them before, but they ask during their visit to the REH where they can buy spectacles, etc. Then they are referred to me, which is really great. (Respondent 16)
·	I: As regards the fact that you are part of the Eye Care Network, do you use it, put a sign on the door: ‘Optician, member of the Eye Care Network’? R: Um, good question. Hardly. I: Why not use it? R: Because it has no effect. I: How do you deduce that? R: Instinctive, advertising is purely instinctive. (Respondent 18)
·	If you ask me what I think needs to be done, then I think health care insurers are keeping out of the way and do not take enough action in this matter. I think that when it comes to eye care, the health care insurers should accept their responsibility. (Respondent 18)
Dutch Healthcare Authority / Health care insurers:	
·	When it comes under the B segment (tariff becomes negotiable) the health care insurer will say: we no longer pay for the part of the DBC delivered by primary care optometrists, because we are already paying those optometrists directly. That is a possibility. Then the Dutch Healthcare Authority does not have to set a price. (The Dutch Healthcare Authority, respondent 27)
·	If the reason for your question is: would we insurers be prepared to contract an optometrist, to agree on a tariff and let him be responsible for this care; that is something I would be prepared to consider. But on the condition that the quality of care is guaranteed, that the Health Care Inspectorate is confident about it, and above all that the referring glaucoma specialists have confidence in it. (Health care insurer, respondent 25)
Patients:	
·	I think it is a bit more reassuring when you stay under your doctor’s care, of course. A specialist is probably a bit more knowledgeable. You're so used to it. (Respondent 24)
·	In some ways, care by a local optometrist might be nicer. The ophthalmologist with his experience and knowledge might see certain things very quickly, though. But I have the impression that at the GFU they have a bit more time for you, they want to know things exactly and are more precise than the doctor.
	But still, if I have the choice between one and the other and they are both of good quality, then I would choose the one that doesn’t cost me anything. (Respondent 23)

This new collaboration put a strain on the REH-OCR collaboration. The optometrists had seen the monitoring of glaucoma patients as opportunity to compete with optical chains because they could not compete with them on the price of eyewear. On the other hand, some primary care optometrists were unsure about the competitive advantage of membership in the REH’s eye care network. Despite their belief that monitoring glaucoma patients would provide work diversity, whether it would result in additional clients or income was unclear.

The combination of an unclear effect of eye care network membership with the absence of a separate reimbursement tariff for optometric examinations rendered the monitoring of glaucoma patients financially unattractive for primary care optometrists.

In the absence of a reimbursement tariff for an optometric examination, most patients did not choose primary care, because they would have to pay (directly or indirectly) for care that otherwise was reimbursable. Besides, some patients claimed that they relied more on the glaucoma specialists than the primary care optometrists. Such considerations outweighed the advantages of the optometrists’ care, such as flexible appointments and proximity to patients’ homes.

Health insurers could mediate between primary care optometrists and the REH to realize shared care and provide an appropriate reimbursement tariff, but were at the moment of evaluation in 2008 reluctant to initiate discussions. They claimed, however, that if the REH could guarantee the quality of care and if physicians supported the substitution, they would seriously consider recommending reimbursement of primary care optometric examinations to the Dutch Health Care Insurance Board.

The Dutch Healthcare Authority mentioned another problem in this regard: the realization of a tariff for an optometric examination would result in additional costs for health insurers and their clients because such tasks (monitoring stable glaucoma patients) were also financed for hospital-based glaucoma specialists. This was, however, a temporary problem as the reimbursement of glaucoma monitoring by glaucoma specialists became negotiable in 2009 [44].

### Power positions and level of interest

In Table 5 we summarised the stakeholder positions by mapping their willingness to participate in the task substitution and their power positions.

**Table 5:** Summary of the stakeholder positions

**Stakeholders**	**Support / opposition**	**Power-position**
**REH management**	Moderate support	Medium
**Glaucoma specialists**	Strong opposition	High
**GFU employees**	Strong opposition	Low
**Primary care optometrists**	Strong support	Low
**Patients**	Neutral	Medium
**Health insurers / Nza**	Neutral	High

The REH management initially wanted to transfer visits of stable glaucoma patients to primary care optometrists with the conditional support of the Dutch Healthcare Authority, patients, and health insurers. The conditions of the Dutch Healthcare Authority included accessibility and affordability of care (Table 6). Although patients saw benefits of the task substitution, like faster and more flexible appointments and shorter travelling times, they were not likely to visit primary care optometrists if they had to pay for care that would have been otherwise reimbursed (Table 6). The reimbursement could be accelerated by the health insurers, but they stood with the patients, arguing that they were only willing to cooperate if glaucoma specialists and the Health Care Inspectorate were fully behind the task substitution.

**Table 6:** Quotations ‘Power positions and level of interest’

**Stakeholder**	**Quotation**
Management REH:	
·	With respect to glaucoma care, we have started to investigate whether apart of the activities that take place here could be substituted to optometrists who are closer to the patient’s home. (Respondent 2)
·	I: What is your opinion about substitution of eye care to other professionals?
	R: If we did not agree, we would not put so much energy into it. (Respondent 1)
The Dutch Healthcare Authority / Health care insurers:	
·	It looks like it would be more accessible than going to see a doctor. In that respect it seems to be in the interests of the patient. It seems like a good development. (The Dutch Healthcare Authority, respondent 27)
Patients:	
·	Yes, I thought it was safe, so I thought, well, if the doctor says so. I simply trust him, so you go along with it. (Respondent 21)
·	I think it is a bit more reassuring when you stay under your doctor’s care, of course. A specialist is probably a bit more knowledgeable. You're so used to it. (Respondent 24)
Primary care optometrists:	
·	Yes, there is a professional group, but I never hear anything about it. A great deal would have to be done there. So I'm afraid that that is also a factor. (Respondent 18)
Glaucoma specialists:	
·	So you need to move forward with small steps, take the lead yourself. Then you have to get clear results which you can show, and once you have these, you can gain the trust of others to do it. (Respondent 7)

Only one glaucoma specialist preferred the situation of having stable glaucoma patients monitored by primary care optometrists. The remaining three specialists and most GFU employees were not in favor.

The OCR optometrists supported the task substitution of monitoring stable glaucoma patients at their shops because the task substitution would improve their professional image and strengthen their competitive positions relative to other optometrists. Their power position, however, was relatively weak due to the OCR’s low degree of organization and the self-employed state of most of their optometrists, unlike, for example, large optical chains. The role of the primary care optometrists was therefore relatively passive (Table 6).

Stakeholders holding strong power positions were glaucoma specialists and health insurers. Although the health insurers supported the idea of task substitution, they did not intend to initiate discussions about the establishment of tariffs for primary care optometrists unless the REH could guarantee quality of care. Besides, insurers believed the active support of the glaucoma specialists important, as did most patients. Thus, the substitution of person and setting would not succeed in the short term. Even the most enthusiastic glaucoma specialist affirmed this (Table 6).

## Discussion

Transferring the monitoring of stable glaucoma patients to primary care optometrists did not seem feasible in the Rotterdam area in the period 2004 to 2011, despite other studies indicating that (primary care) optometrists can provide high-quality care [8-20,37]. The implementation turned out to be the stumbling block as the involved professionals quarrelled over professional boundaries and work domains, they disagreed on the capabilities of primary care optometrists, the assumed returns on investment were unclear after all, and power positions favoured the status quo.

The three theoretical perspectives used in our case study align very well with the implementation literature that broadly acknowledges that implementation of an innovation can be difficult and a well-designed implementation process is critical [33,38,39,45-49].

Our results can be explained by classical theories on implementation, financial incentives, and stakeholder interests. From implementation theories, for example, it could be expected that ophthalmic examination by primary optometrists would be difficult without reimbursement, because the current situation (no reimbursement) differed from the desired situation (with reimbursement) [46]. A second example is the failure to enhance ophthalmologists’ perceived benefit of the innovation by staffing the GFU with primary optometrists at the outset, because that would have reduced the uncertainty about the future situation, which is very important to let the task substitution succeed [33].

This study also taught us two theoretical lessons that might influence the implementation literature. First, the findings highlighted the merit of connecting the implementation literature with sociological theories on professionalism, which is also acknowledged by Adler *et al*. (2009). A key notion for understanding the feasibility of the task substitution was recognizing the path dependency of the professionalization of the occupations involved. There was indeed a window of opportunity for the task substitution at the outset because ophthalmologists were pressured to make professional relationships and domains more fluid. The intermediate step of the GFU fixed the professionalization processes, and the pressures were sufficiently relieved. Thus, being aware of (local) professionalization processes combined with good timing seem crucial for successfully transferring tasks from one professional group to another. Combining implementation literature with sociological theories on professionalism [38-42], we can say in general that it is crucial to involve the substituting professionals early on to ensure that stakeholders see the change as a normal step in the professionalization of the substituting professionals.

Second, the study underscores the importance of time element; windows of opportunity may disappear over time. In Rotterdam, the accumulation of (local) dynamics proved the assumed benefits of the task substitution wrong in the end. The assumption that cooperation with primary care optometrists would increase the inflow of new patients appeared incorrect. Besides, the assumed financial benefits were unclear. Because only hospital visits were reimbursed, primary care optometrists had to monitor stable glaucoma patients for free, charge patients for the examination, or organise a payment by the REH, all of which reduced its benefits. The last possibility would be to create a separate tariff for monitoring glaucoma patients by primary care optometrists, but the power positions of key stakeholders did not support this. Implementation scientists thus should not underestimate (the effect of) changing aims and interests of the different stakeholders over time because they often do change. It is therefore crucial to make the right decisions at the right time to obtain the expected result.

## Conclusions

National and local factors hamper transferring the responsibility to monitor stable glaucoma patients from the REH’s GFU to primary care optometrists in an outpatient setting. Task substitution in person and in setting is therefore not feasible in the short term in the Rotterdam area.

Unlike the primary care optometrists, most hospital-based glaucoma specialists over time were unwilling to collaborate in the scheme. Moreover, the REH management’s enthusiasm for the task substitution waned when its aim shifted from cooperating with primary care optometrists towards increasing the inflow of new patients, as the demand could be met with the newly established and successful hospital-based GFU.

Policy makers considering substituting tasks to lesser trained professionals as well as substituting the delivery of services from a hospital to a primary care setting should carefully think about the implementation process, especially when they decide to implement task substitution in separate steps. Our case study demonstrates that professional, financial, managerial, and political factors all play a role in rendering task substitution feasible and that consolidating task substitution within a hospital setting will freeze the opportunity to transfer to a primary care setting. Recognizing a restricted window of opportunity in the implementation of task substitution is critical.

## Abbreviations

REH: Rotterdam Eye Hospital; GFU: Glaucoma Follow-up Unit; OCR: Collective of Optometrists in Rijnmond Region; TG-project: Transmural Glaucoma-project; TOZ-project: Transmural eye care for all indications.

## Competing interests

The authors declare that they have no competing interests.

## Authors’ contributions

KHG, TP, EvS and MK conducted the interviews and drafted the manuscript. HL, MK and NK provided feedback on our interpretation of the data and critically appraised the research process. All authors have read and approved the final manuscript.

## Supplementary Material

Additional file 1 Background information about the hospital, glaucoma and GFU working procedure and protocol. Click here for file

Additional file 2 Topic list. Description of data: Topic list used for interviewing. Click here for file
