# Supplementary material for: Why a successful task substitution in glaucoma care could not be transferred from a hospital setting to a primary care setting: a qualitative study
Source: Implement Sci. 2013 Jan 25;8:14. doi: 10.1186/1748-5908-8-14 (PMC3576268; doi:10.1186/1748-5908-8-14)
Supplement: Additional file 1 — Background information about the hospital, glaucoma and GFU working procedure and protocol. [file 1748-5908-8-14-S1.doc]

**Additional file 1. Background information about the hospital, glaucoma and GFU working procedure and protocol**

The Rotterdam Eye Hospital is a Centre of Excellence that provides high level medical, paramedical and nursing care, and that pays much attention to the transfer of knowledge. In 2009, the number of outpatient visits was 138,311 and the number of hospitalizations was 1,048.

Glaucoma is a group of eye diseases characterised by damage to the optic nerve that causes gradual, irreversible visual field loss. It is often related to age and high intraocular pressure, and care is currently provided by general ophthalmologists and glaucoma specialists. It calls for a tailored approach for each individual patient to slow down or halt the natural course of the disease. Monitoring patients by optometrists or ophthalmic assistants might be sufficient for stable glaucoma patients who are regulated correctly by ophthalmologists or patients at risk for glaucoma.

Those patients were referred to the GFU by their treating ophthalmologist. As long as patients were stable according to specific criteria for back referral, they visited the GFU twice followed by a visit to the glaucoma specialist or resident. If the patient was not stable according to these criteria, the patient was seen by a glaucoma specialist earlier. The GFU employees performed the following activities: ask a short history, determine the IOP and Snellen visual acuity and make GDx images. In case of moderate to advanced visual field damage or at doctor’s request, they performed an additional yearly HFA (Humphrey Field Analyser, standard 24-2 test algorithm; Carl Zeiss Meditec, Dublin, CA, USA).
