# Supplementary material for: Why a successful task substitution in glaucoma care could not be transferred from a hospital setting to a primary care setting: a qualitative study
Source: Implement Sci. 2013 Jan 25;8:14. doi: 10.1186/1748-5908-8-14 (PMC3576268; doi:10.1186/1748-5908-8-14)
Supplement: Additional file 2 — Topic list. Description of data: Topic list used for interviewing. [file 1748-5908-8-14-S2.doc]

**Additional file 2. Topic list**

**Introduction**

Introduction of study, the interviewees, and the participant

1. What is your attitude towards task substitution in general and more specifically in glaucoma care?
2. What are, according to you, the advantages and disadvantages of task substitution?
3. How would you define and picture the task substitution in glaucoma care in the ideal situation?
4. What basis criteria need to be fulfilled? To what extent have they already been fulfilled? And if applicable: how can you accomplish that?
5. For what reasons would you (not) cooperate to let primary care optometrists become substitutes for glaucoma specialists in monitoring stable glaucoma patients?
6. What would you (or your organization) do to make sure that the task substitution will (not) be established or to increase or decrease the chance of its successful implementation?
7. Which adjustments to the current health care system are required to establish the proposed task substitution?
8. Are there any other issues you would like to discuss?
